# Severity of hypertensive retinopathy and its determinants among adult patients attending ophthalmic centers in Northern Ethiopia: A multicenter cross-sectional study

**DOI:** 10.1186/s12886-026-04846-1

**Published:** 2026-04-21

**Authors:** Yitayal Abebe Gudayneh, Abebech Tewabe Gelaye, Sisay Eshete Tadesse

**Affiliations:** 1 Department of Opthalmology, Boru Meda General Hospital, Dessie, Ethiopia; 2 Department of Clinical Pharmacy, University of Gondar College of Medicine and Health Science, Gondar, Ethiopia; 3 School of Public Health, Wollo University, College of Medicine and Health Science, Dessie, Ethiopia

**Keywords:** Hypertensive retinopathy, Adult systemic hypertensive, Severity, Northern Ethiopia

## Abstract

**Introduction:**

Hypertensive retinopathy reflects systemic vascular damage and serves as an indicator of end-organ involvement among patients with systemic hypertension. Despite its clinical importance, evidence on disease severity and its determinants in low- and middle-income settings, including Northern Ethiopia, remains limited.

**Objective:**

To assess the severity of hypertensive retinopathy and its determinants among adult patients attending ophthalmic centers in Northern Ethiopia in 2025.

**Methods:**

A multicenter, institutional-based cross-sectional study was conducted among adult patients with confirmed hypertensive retinopathy attending ophthalmic centers of Amhara region comprehensive specialized hospitals from May 7 to November 7, 2025. Data were collected using KoboToolbox and exported to STATA version 17 for analysis. Bivariable and multivariable ordinal logistic regression analysis were performed to identify factors associated with disease severity of hypertensive retinopathy. Variables with a p-value < 0.05 in the multivariable analysis were considered statistically significant.

**Results:**

A total of 400 hypertension adults with confirmatory hypertensive retinopathy cases achieving a response rate of 96.62% were involved under the study. History of cataract surgery (AOR = 2.19; 95 CI: 1.32–3.61), Diabetes mellitus (AOR = 1.61; 95% CI: 1.05–2.47), and chronic kidney disease (AOR = 3.16; 95% CI: 1.82–5.48) were significantly associated however regular hypertension check-up (AOR = 0.47; 95% CI: 0.27–0.80) was protective.

**Conclusion:**

Severity of hypertensive retinopathy were significantly associated with history of cataract surgery, diabetes mellitus and chronic kidney disease while regular hypertension follow-up was protective highlighting the importance of integrated clinical evaluation and routine retinal screening in hypertension patient care.

## Introduction

Hypertension (HTN) is a non-communicable medical condition in which the blood pressure (BP) in the arteries is consistently high [1]. Currently, over 1.4 billion adults worldwide are living with HTN, making it one of the most common chronic conditions globally and a major risk factor for cardiovascular disease, including heart failure. An estimated two-thirds of these individuals reside in low- and middle-income countries, where limited awareness, delayed diagnosis, and suboptimal treatment contribute to a high prevalence of uncontrolled BP and subsequent target organ damage(TOD) [2–4]. In these settings, inadequate awareness, late diagnosis, and poor treatment adherence contribute to uncontrolled BP and subsequent target organ damage like heart, kidney, brain and the retina causing hypertensive retinopathy (HR) [4].

HR is a well-recognized microvascular complication of systemic HTN, reflecting structural and functional alterations in the retinal vasculature due to prolonged elevated BP [5]. Clinically, HR is characterized by retinal arteriolar narrowing, arteriovenous crossing changes, hemorrhages, exudates, and in advanced stages, optic disc edema [6]. Beyond its ocular implications, HR serves as an important predictor of systemic vascular disease and is strongly associated with increased risks of stroke, coronary artery disease, and renal dysfunction [6].

The severity of HR is closely linked to the duration and level of HTN, as well as other systemic and behavioral factors. Severe forms of HR are particularly concerning because they are associated with end-organ damage and poorer clinical outcomes [6]. Studies have shown that factors such as older age, poorly controlled BP, long duration of HTN, comorbid diabetes mellitus, dyslipidemia, and poor adherence to antihypertensive therapy significantly contribute to both the development and progression of HR [7, 8]. In addition, lifestyle factors and access to healthcare services further influence disease severity, especially in resource-limited settings [9].

Globally, the prevalence and severity of HR vary widely depending on population characteristics and diagnostic criteria, with higher rates reported among individual [7, 8]. In sub-Saharan Africa, where HTN is increasingly prevalent and often poorly managed, the burden of HR is substantial. Recent studies from Ethiopia have reported a high prevalence of HR among hypertensive patients, with a considerable proportion presenting with moderate to severe disease [8]. For instance, a multicenter study in Northwest Ethiopia found that more than half of hypertensive patients had evidence of retinopathy, with severe stages associated with uncontrolled BP and comorbid conditions [8].

Despite this growing burden, there is limited evidence focusing specifically on the determinants of disease severity among patients already diagnosed with HR. Understanding the factors associated with severity of HR is essential for early identification of high-risk patients, prevention of visual impairment, and reduction of systemic complications. However, in Ethiopia, particularly in Northern regions, evidence on determinants of severity among this specific population remains scarce. Therefore, this study aimed to assess the disease severity and its determinants among adult patients with HR attending ophthalmic centers in Northern Ethiopia.

## Methods and materials

### Study design

#### Study area and period

A multicenter, hospital-based cross-sectional study was carried out in Northern Ethiopia in which eight comprehensive and specialized hospitals are available. These are Woldia, Dessie, Debre Berhan, University of Gondar, Felege Hiwot, Debre Markos, Debre Tabor, and Tibebe Ghion. The study was conducted specifically in the ophthalmology centers of five of these hospitals—University of Gondar, Felege Hiwot, Debre Markos, Debre Tabor, and Tibebe Ghion—from May 7 to November 7, 2025. All selected hospitals are equipped with well-established ophthalmic centers staffed by specialized ophthalmologic professionals.

### Source and study population

#### Source population

The source population comprised all adult patients with systemic HTN who were undergoing ophthalmologic follow-up care and had been diagnosed with HR by ophthalmologists at the ophthalmic centers of comprehensive and specialized hospitals in Northern Ethiopia.

#### Study population

The study population consisted of all adult patients with systemic HTN who were receiving ophthalmologic follow-up care with HR diagnosis by ophthalmologists at the ophthalmic centers of the selected comprehensive and specialized hospitals during the study period.

### Inclusion and exclusion criteria

All adult patients with systemic HR who were receiving ophthalmologic follow-up care and had been diagnosed with HR by ophthalmologists at ophthalmic centers of specialized hospitals in Northern Ethiopia, and who had clear ocular media for retinal examinations were included under the study. Participants with conditions that obscured posterior segment evaluation—such as corneal opacity or ulcer, lens opacity or vitreous opacity—were excluded.

### Sample size determination

#### Objective one

The sample size was calculated by using single population proportion formula by considering the following assumptions:


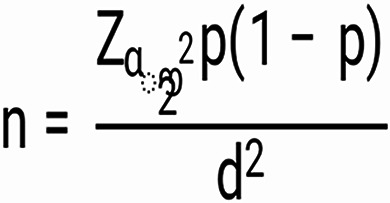


Where,

n = the required sample size,

Z= Value of z statistic at 95% confidence level = 1.96,

P= expected proportion to HR among adult hypertensive patients were 57.47% [5]. This study was conducted in Northwest Ethiopia.

d = margin of error, which was 5% (0.05), therefore, the calculated sample size was 376.

#### Objective two(factor)

Uncontrolled BP, HTN treatment [10] and cardiovascular(heart) disease [8] were used in which they were statistically significant factors for HR under the studies and were used to calculate the sample size of objective two through Epidemiological Information (EPI INFO) 7 computer software (Table 1).

Therefore, the sample size calculated for the first objective was larger than that of the second objective. After accounting for a 10% non-response rate, the final sample size was 414.

#### Sampling technique and procedures

A systematic random sampling technique was employed. Of the eight comprehensive specialized hospitals in the Northern Ethiopia, five were selected using a lottery method. The total sample size was then proportionally allocated based on the number of patients attending the ophthalmic centers of these hospitals.

From the registration records of the five selected hospitals, on average 1,314 adult HR patients had a history of ophthalmic follow-up over the past three years during the same data collection period (May 7 to November 7 in 2022, 2023, and 2024). Using this information, proportional allocation was performed across the hospitals.

**Fig. 1:**
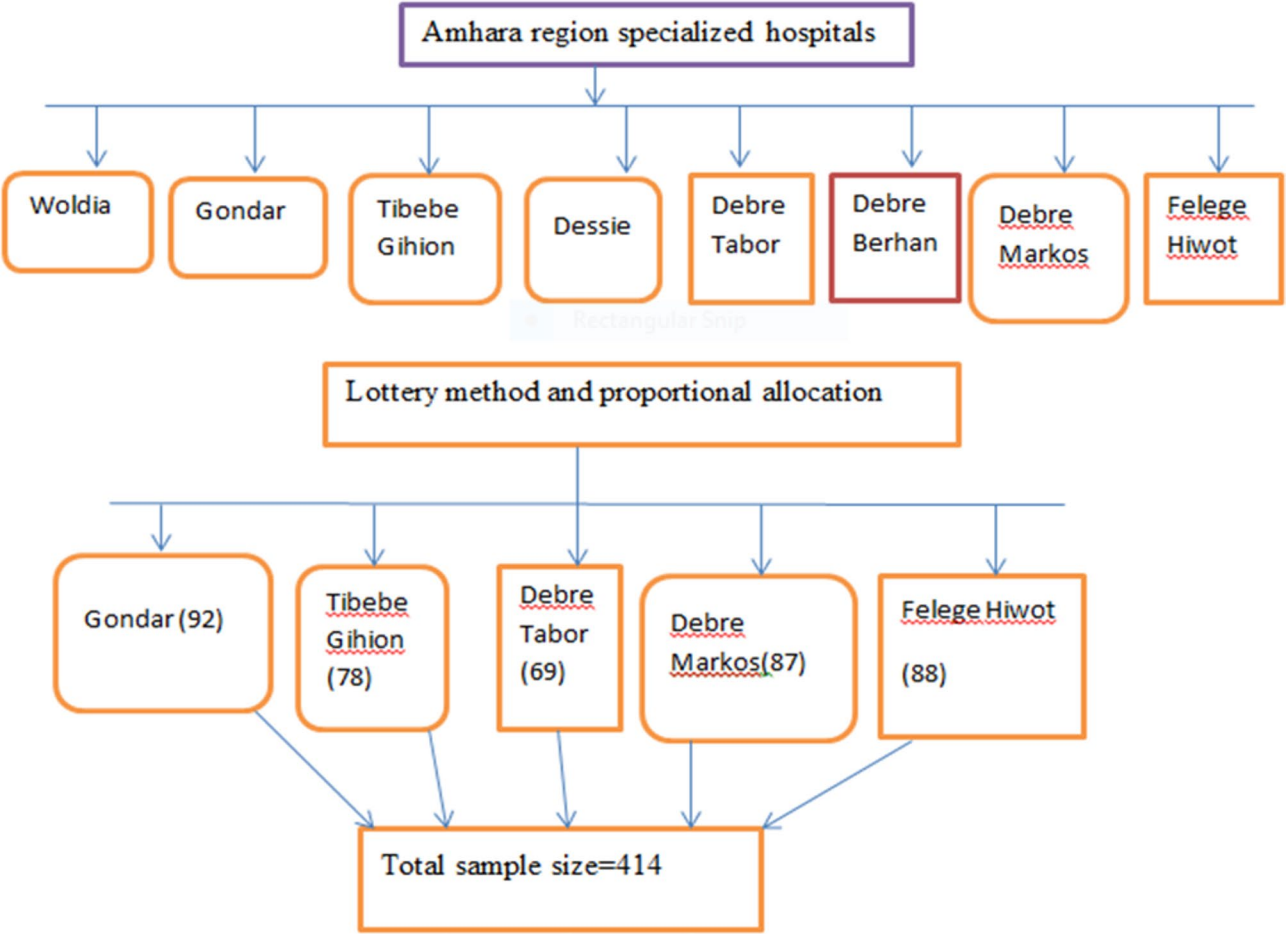
Sampling procedure and sampling techniques of HR study participants attending Ophthalmic Centers in Northern Ethiopia, 2025

The sampling interval (k) was calculated as (k = N/*n* = 1314/414 = 3.17), which was approximated to 3. The first participant was selected by a lottery method from the first three eligible individuals, and subsequently, every third participant was included in the study (Fig. 1).

### Dependent variable: HR

#### Independent variables

##### Socio-demographic variables

Age, sex, residence, marital status, educational status, occupational status, health insurance, and family history of HTN.

##### Behavioral factors

Cigarette smoking, physical exercise, checking BP regularly, and adherence to hypertensive medication.

##### Systemic comorbidities factors include

Atherosclerosis, diabetes mellitus, chronic heart disease, chronic kidney disease (CKD), dementia, cognitive impairment, and dyslipidemia.

##### Clinical factors

Duration of HTN since diagnosis, uncontrolled HTN, body mass index, duration of HTN medication,

#### Operational definitions

HR was diagnosed using a + 90D Volk lens under mydriasis with tropicamide 1% eye drops. It was categorized as HR positive if at least one of the following clinical findings was observed in at least one eye: detectable arterial narrowing, arteriovenous nicking, retinal hemorrhage and/or exudates, disc swelling (papilledema), silver wiring, vascular tortuosity [11, 12]. Additionally, HR was classified into stages: - Stage 1: Widening of the arteriole light reflex.

- Stage 2: Stage 1 + arteriovenous crossing sign.
- Stage 3: Copper wiring of arterioles (copper-colored arteriole light reflex).
- Stage 4: Silver wiring of arterioles (silver-colored arteriole light reflex) using modified Scheie Classification.

##### History of eye examination

defined as having at least one eye examination within one year following a diagnosis of HTN, before the data collection period [13].

##### Physical exercise

This can be defined poor; when regular exercise was less than 150 min (3–5 days) of work per week and it was considered to have good regular exercise when greater than 150 min(≥ 5days) of work per week [14].

##### Alcohol drinking

The level of alcohol consumption was categorized as current alcohol users if the study participants took alcoholic drinks within 30 days preceding the study; as moderate drinkers when participants consumed standard of two drinks on a single occasion for men, one drinks on a single occasion for women and as heavy drinkers if participants consumed standard of five or more drinks on a single occasion or twenty or more drinks per week for men, four or more drinks on a single occasion or fifteen or more drinks per week for women [15].

##### Current levels of HTN

Grouped under uncontrolled when (systolic BP ≥ 140mmHg or diastolic BP ≥ 90mmHg) [16].

##### CKD

Is defined when estimated glomerular filtration rate (eGFR) < 60 mL/min/1.73 m² for ≥ 3 months, or evidence of kidney damage (proteinuria, structural abnormalities [17].

##### Diabetes mellitus

was defined as a participant having a prior diagnosis of diabetes by a health professional, current use of antidiabetic medication, or a fasting blood glucose level ≥ 126 mg/dL (7.0 mmol/L) or random blood glucose ≥ 200 mg/dL (11.1 mmol/L) during the study period [18].

##### Visual acuity

Was categorized as normal visual acuity (if the presenting visual acuity or PVA is better than 6/12), mild visual impairment (if PVA is < 6/12 to ≥ 6/18), moderate visual impairment (if PVA is < 6/18 to ≥ 6/60), severe visual impairment (if PVA is < 6/60 to ≥ 3/60), and blind (if PVA is less than 3/60). These categories are based on the 11th definition of visual impairment by the International Classification of Disease [19].

#### Data collection technique

A structured questionnaire was adapted from previously published similar studies [20, 21] and a standardized checklist was developed. Comprehensive eye examinations were conducted for each study participant. Presenting distance visual acuity was assessed using a Snellen acuity chart at a distance of 6–3 m under optimal room illumination. Anterior segment examination was performed initially, followed by posterior segment evaluation after pupillary dilation with 1% tropicamide. The posterior segment was examined using a slit-lamp biomicroscope with a 90-diopter Volk lens to diagnose HR and determine its severity. BP was measured for all study participants. Sociodemographic characteristics were collected through face-to-face interviews, while other relevant medical information was obtained from patients’ medical records. Laboratory profiles of the study participants were obtained either through review of medical records, if the investigations had been performed within the preceding 3–4 months, or through newly requested tests when recent results were unavailable.

#### Data quality assurance

To maintain the quality of the study, the data collection instruments were pretested on 5% of the sample at Debre Tabor Comprehensive Specialized Hospital before 3-months of the data collection. The tools, originally prepared in English, were translated into Amharic and translated back to English to facilitate communication and ensure clarity for participants. Data collectors received training from the principal investigator on the overall data collection procedures, and close supervision was provided throughout the process. Additionally, completed questionnaires were reviewed daily for accuracy and completeness to ensure overall data quality.

#### Data processing and analysis

All the necessary information collected from the study participants were entered into kobo toolbox and then transferred to STATA version 17 for analysis. Proportions and summary statistics such as median and standard deviation were calculated for the descriptive data. Bivariable followed by a multivariable ordinal logistic regression was fitted to determine factors associated with severity of HR. Variables with a P-value less than 0.25 in the bivariable analysis were entered into a multivariable logistic regression. The strength of the association between dependent and independent variables was expressed by an adjusted odds ratio (AOR) with a 95% confidence interval (CI). Variables having p-values of less than 0.05 in multivariable logistic regression analysis were declared as associated factors of HR. Multi-collinearity was checked using the variance inflation factor and tolerance and no significant relationship was detected between the predictor variables (VIF = 2.25).

## Results

### Socio-demographic and behavioral characteristics of the study participants

A total of 400 participants were enrolled in the study, yielding a response rate of 96.62%. Of these participants, 223 (55.75%) were male, 202 (50.50%) were aged ≥ 50 years, 72 (18%) had a family history of hypertension, and 75 (18.12%) were literate. Additionally, 164 participants (41%) underwent regular eye examinations, 164 (41%) reported good exercise habits, and 83% regularly monitored their blood pressure after being diagnosed with HTN (Table 2).

### Clinical and systemic characteristics

All study participants were evaluated for systemic comorbidities in addition to HR. Among them, 211 (52.75%) had uncontrolled BP, and 267 (66.75%) had been living with HTN for ≥ 5 years. CKD was present in 77 participants (19.25%), chronic heart disease in 132 (33%), stroke in 105 (26.25%), and 26 participants (6.5%) were diagnosed with atherosclerosis (Table 3).

### Laboratory profiles

Among the study participants, 245 (61.4%) had total cholesterol levels ≤ 200 mg/dl, while 155 (38.6%) had elevated levels. Similarly, 221 (55.25%) participants had triglyceride levels ≤ 150 mg/dl, whereas 179 (44.75%) had elevated values while 237 (59.25%) participants had low-density lipoprotein (LDL) levels ≤ 160 mg/dl and 163 (40.75%) had higher levels. Most participants had normal renal function markers: 333 (83.25%) had serum creatinine ≤ 1.1 mg/dl and 339 (84.75%) had blood urea nitrogen (BUN) ≤ 50 mg/dl. Elevated serum creatinine and BUN levels were observed in 16.75% and 15.25% of participants, respectively. Additionally, 342 (85.5%) participants had serum uric acid levels ≤ 7.1 mg/dl, while 58 (14.5%) had elevated levels (Table 4)**.**

### Severity distribution of hypertensive retinopathy

**Fig. 2:**
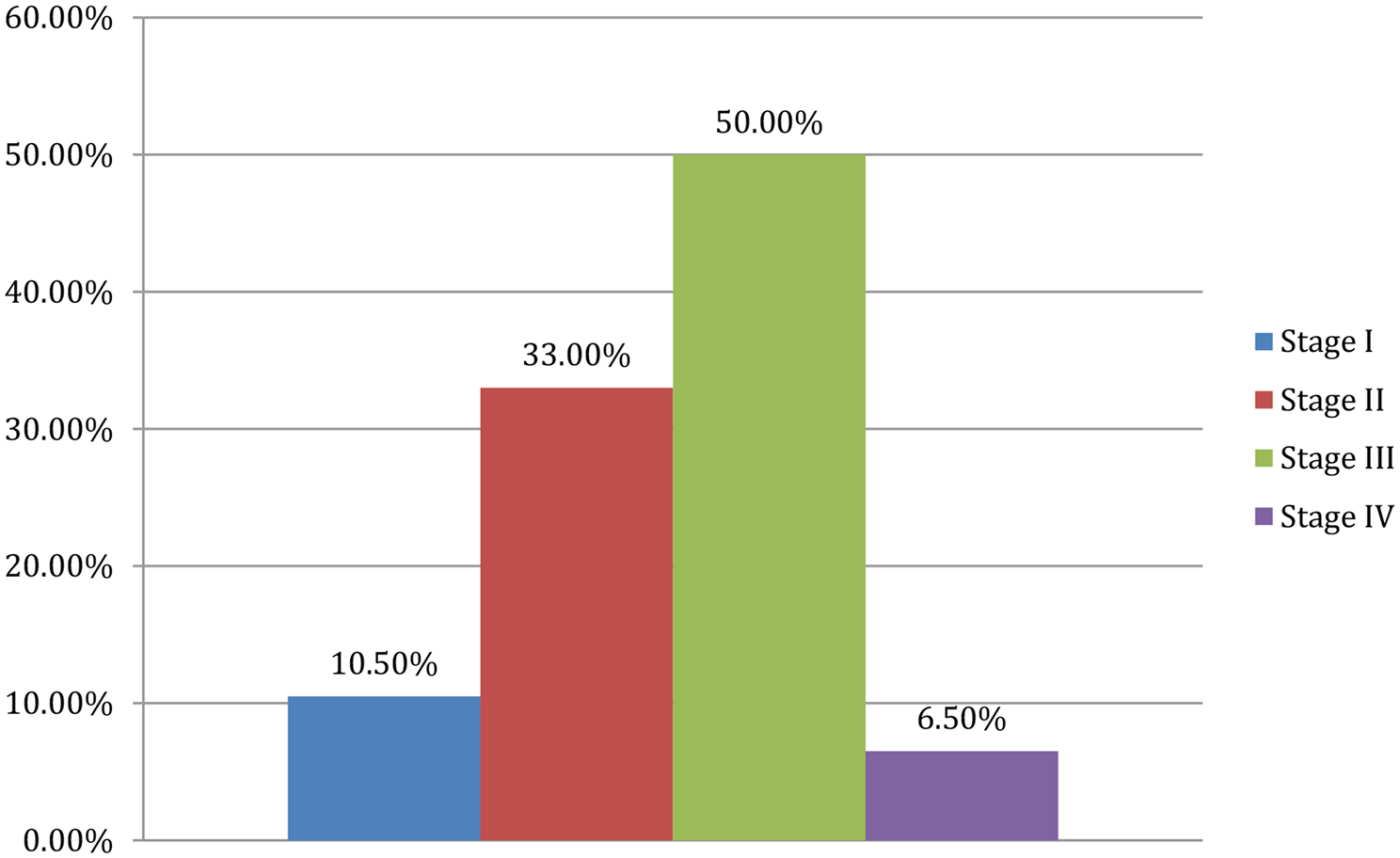
Proportions of hypertensive retinopathy by severity among adult patients attending ophthalmic centers in Northern Ethiopia, 2025

Of the 400 participants, stage III HR was the most common (200, 50%), followed by stage II (132, 33.0%) (Fig. 2).

### Factors associated with severity of HR

This study employed an ordinal logistic regression model to identify factors associated with severity of HR. The model estimates the proportional odds of being in a higher severity category versus all lower categories combined. During bivariable ordinal logistic regression, history of ocular surgery, physician advice for eye check-up, history of diabetes mellitus, CKD, sex, regular HTN checkup, alcohol drinking and eye examination after HTN diagnosis were eligible for final model. In multivariable ordinal logistic regression analysis only history of cataract surgery, history of diabetes mellitus, CKD and regular HTN checkup were remained significant factors associated with severity of HR.

The overall model was statistically significant (LR χ² = 58.73, *p* < 0.001), indicating that the included covariates collectively contribute to explaining variation in HR severity.

Participants with a history of cataract surgery were two times more likely to have severe HR than those with no history of ocular surgery (AOR = 2.19; 95% CI: 1.32–3.61).

Participants with diabetes mellitus were 61% more likely to have severe HR compared to those with no diabetes mellitus (AOR = 1.61; 95% CI: 1.05–2.47).

This study revealed that participants with regular HTN follow-up were 53% less likely to have severe HR than those who with no regular follow-up (AOR = 0.47; 95% CI: 0.27–0.80).

Participants with history of CKD were 3times more likely to have severe hypertensive retinopathy than those with no history of CKD (AOR = 3.16, 95% CI: 1.82–5.48) (Table 5).

## Discussion

The aim of this study was to assess the stages of HR and associated factors among hypertensive patients diagnosed with HR during their ophthalmic follow-up. This study demonstrates that the severity of HR is strongly associated with history of diabetes mellitus, CKD, and ocular surgery and regular HTN follow-up.

The present study revealed that participants with history of diabetes mellitus had a 61% higher likelihood of progressing to more severe stages of HR compared to those non-diabetic patients. The result is consistent with recent study evidence from a multicenter study in Northwest Ethiopia [8], Tanzania [22] and England [23]. This consistency between findings strengthens the evidence that diabetes mellitus is a key contributor not only to the presence but also to the severity and progression of hypertensive retinal vascular damages.

The biological reasons of this finding could be as chronic hyperglycemia in diabetes leads to endothelial dysfunction, oxidative stress, and thickening of the capillary basement membrane, all of which compromise retinal microcirculation damages. When these conditions combined with HTN, the processes resulted in accelerated arteriolar narrowing, increased vascular permeability, and ischemic retinal injury, thereby promoting more advanced stages of retinopathy [24].

Evidence suggests that individuals with coexisting HTN and diabetes exhibit more pronounced microvascular dysfunction across multiple organs, including the retina could demonstrate parallel progression of retinal and other microvascular complications in diabetic hypertensive patients resulting more severe HR [25].

However, the observed association between diabetic mellitus and HR severity should be interpreted in light of the study’s cross-sectional design, which limits causal inference. Future longitudinal studies are warranted to better elaborate the temporal and causal pathways linking diabetes to the progression of HR severity in resource limited setting including Ethiopia.

This study confirmed that participants with a history of ocular surgery had more than a two-fold increased likelihood of being in a higher severity of HR compared to those without such history ocular surgery. The study result is consistent with other studies conducted in Nepal done by Bhaktapur Retina Study [26] and Thailand [27]. This could be due to: First, a history of ocular surgery often reflects underlying ocular pathology, such as cataract, glaucoma, or retinal disorders, which may already compromise retinal integrity. Such pre-existing structural or vascular abnormalities could increase susceptibility to hypertensive damage and increased its severity [28]. Second, surgical procedures may induce alterations in ocular blood flow and retinal autoregulation, potentially exacerbating the effects of chronic hypertension on retinal microvasculature resulting severity of retinopathy [29, 30]. Third, disruption of the blood-retinal barrier and postoperative inflammatory responses may further contribute to microvascular instability of the retina resulting in more severe HR [31].

Another reasonable could be surveillance (detection) bias in which real case and time individuals who have undergone ocular surgery are more likely to have frequent ophthalmologic follow-up which increases the probability of detecting HR, particularly at more advanced stages of the disease [32, 33]. This is phenomenon is consistent with observational and epidemiological studies, where increased healthcare contact leads to higher detection rates of subclinical or progressive disease [34, 35]. On the contrary, the observed association may partly reflect reverse causality contradicting the study result, whereby patients with more severe HR or symptomatic retinal disease are more likely to require surgical intervention, rather surgery itself contributing directly to disease progression and severity of this vascular disease [36]. Such bidirectional relationships are a recognized limitation of cross-sectional studies and have been noted in research on retinal and systemic vascular conditions. Lastly, from a clinical standpoint, this finding highlights the need for closer retinal monitoring among hypertensive patients especially with a history of ocular surgery, as they may represent a high-risk subgroup for advanced disease of HR. Longitudinal studies are needed to clarify HR disease severity and cataract surgery risk in low- and middle-resource settings, including Ethiopia.

This study confirmed that participants with a history of chronic kidney disease (CKD) had 3.16 times higher odds of severe HR compared to those without CKD. This finding is consistent with the study conducted in China, Nepal and Italy an emerging evidence demonstrating that CKD is strongly associated with the severe HR [24, 31, 37]. This could be due to markers of renal impairment, including reduced glomerular filtration rate and albuminuria, are significantly correlated with retinal vascular abnormalities and progressive retinopathy [25, 38].

Another reason could be the shared similar embryological origins and microvascular structures, making them particularly susceptible to chronic hemodynamic stress and endothelial dysfunction in which CKD contribute to systemic vascular injury through multiple mechanisms of actions which includes persistent inflammation, oxidative stress, and impaired endothelial function. These processes lead to arteriolar narrowing, increased vascular permeability, and microvascular rarefaction, which are hallmark features of HR. In addition, dysregulation of the renin–angiotensin–aldosterone system in CKD further exacerbates vascular damage and accelerates retinal microcirculatory impairment [39]. The observed association could reflect parallel progression of HR, a recognized manifestation of systemic TOD, and renal disease. Large-scale observational studies have consistently shown that advancing stages of CKD are associated with more severe grades of retinopathy, supporting a shared microvascular disease pathway [24, 31].

From a clinical and public health perspective of low resource setting including Ethiopia, this study finding highlights the importance of integrated screening and management strategies of HR and CKD. Hypertensive patients with CKD should be prioritized for regular retinal examination, as they represent a high-risk group for vision-threatening complications. Thus early identification and coordinated management of both renal and retinal involvement may improve overall vascular outcomes and reduce morbidity among hypertensions.

This study revealed that participants who had regular hypertension follow-up exhibited a 53% reduction in the odds of severe HR compared to those without regular follow-up. The finding highlights the critical role of continuity of HTN care in mitigating the progression of hypertensive microvascular complications including HR.

This study result is supported by other previous studies conducted in Northwest Ethiopia [8] and a Singapore [40]. This could be due to Patients who adhere to routine follow-up schedules are more likely to achieve sustained BP control, receive timely medication adjustments, and undergo appropriate screening for hypertensive related complications [41]. Another reason could be Improved BP regulation directly reduces retinal arteriolar damage, vascular leakage, and ischemic changes, thereby slowing the progression of retinopathy [42].

Moreover, regular follow-up visits could provide opportunities for reinforcement of medication adherence, lifestyle modification, and early detection of retinal changes, all of which contribute to improved clinical outcomes and significantly reduce the burden of HTN-related complications by enhancing patient engagement and healthcare delivery efficiency [43]. In addition, consistent follow-up could play a pivotal role in interrupting this trajectory by enabling early intervention there by resulting less severe retinopathy. In resource-limited settings including Ethiopia, however, barriers such as limited access to care, financial constraints, and low awareness may hinder regular follow-up, thereby increasing the risk of advanced retinopathy disease. From a clinical and public health perspective, this finding underscores the need to strengthen chronic care models and follow-up systems, particularly in low- and middle-income countries. Integrating routine retinal screening into hypertension clinics and improving patient retention in care clinic could substantially reduce the incidence of hypertension related vision-threatening complications.

### Clinical implications

Overall, the study findings suggest that HR severity is strongly influenced by systemic disease burden and healthcare utilization patterns. Therefore, clinical management should adopt a risk-based approach, focusing on early identification of high-risk patients, integrating ophthalmic screening into chronic disease care, and strengthening follow-up systems to prevent progression to vision-threatening stages of HR in low and middle income setting including Ethiopia.

## Conclusion

Severity of hypertensive retinopathy were significantly associated with history of cataract surgery, diabetes mellitus and chronic kidney disease while regular hypertension follow-up was protective highlighting the importance of integrated clinical evaluation and routine retinal screening in HTN care.

### Strengths of the study

This study has several strengths including: - A multicenter design enhances the representativeness and generalizability of the findings within the study areas. The use of a relatively large sample size improving the statistical power of the analysis In addition, the study employed standardized ophthalmic assessments to confirm HR, increasing diagnostic reliability. The application of ordinal logistic regression allowed for appropriate modeling of disease severity and adjustment for potential confounders. Furthermore, the study provides context-specific evidence from a low-resource setting, where data on hypertensive retinopathy severity and its determinants are scarce.

### Limitations of the study

This study has some limitations. Being cross-sectional design limits causal inference between the identified factors and severity of hypertensive retinopathy. In addition, the hospital-based setting may introduce selection bias which limits generalizability of the findings to the broader hypertensive population. Since the study was conducted among patients attending ophthalmic centers of comprehensive specialized hospitals, the findings may not be fully generalizable to all hypertensive patients in the community, particularly those who do not seek ophthalmic care.

**Table 1:** Sample size calculation for severity of HR and its determinants with statistically significant factors like chronic heart disease, uncontrolled blood pressure and treatment of HTN

Factor variables	HR					Power and Confidence level	Sample size
	Yes	No	Crude Odd Ratio(COR)	Ratio of unexposed to exposed	outcome Percentage of in the unexposed group		
History of cardiovascular disease			20.33	4	48.11%	80% power 95% confidence level	45
Yes	132	7					
No	268	289					
Uncontrolled BP			0.117	2.96	74%		50
Yes	17	51					
No	20	7					
Treatment of HTN			0.300	1.9	60.9%		116
Yes	23	49					
No	14	9					

N.B: COR: crude odds ratio

**Table 2:** Socio-demographic and behavioral characteristics of HR participants attending ophthalmic centers in Northern Ethiopia, 2025(*n* = 400)

Variable	Category	Frequency (*n*)	Percentage (%)
Sex	Male	223	55.75
	Female	177	44.25
Age Group	≥ 50 years	202	50.50
	< 50 years	198	49.50
Family History of HTN	Yes	72	18.00
	No	328	82.00
Educational Status	Literate	75	18.75
	Illiterate	325	81.25
Regular Eye Examination	Yes	164	41.00
	No	236	59.00
Physical exercise Habit	Good	164	41.00
	Poor	236	59.00
BP check-up Practice	Regular	332	83.00
	Irregular	68	17.00

**Table 3:** Clinical and systemic characteristics of HR participants attending ophthalmic centers in Northern Ethiopia, 2025(*n* = 400)

Variable	Category	Frequency (*n*)	Percentage (%)
HTN Status	Uncontrolled	211	52.75
	Controlled	189	47.25
Duration of HTN	≥ 5 years	267	66.75
	< 5 years	133	33.25
CKD	Yes	77	19.25
	No	323	80.75
Chronic Heart Disease	Yes	132	33.00
	No	268	67.00
History of Stroke	Yes	105	26.25
	No	295	73.75
Atherosclerosis	Yes	26	6.50
	No	374	93.50

**Table 4:** Laboratory profiles of adult HR participants attending ophthalmic centers in Northern Ethiopia, 2025 (*n* = 400)

Variables	Frequency	Percentage
Total cholesterol		
≤ 200 mg/dl	245	61.40
> 200 mg/dl	155	38.60
Triglyceride		
≤ 150 mg/dl	221	55.25
> 150 mg/dl	179	44.75
Low density lipoprotein		
≤ 160 mg/dl	237	59.25
> 160 mg/dl	163	40.75
Serum creatinine (in mg/dl)		
≤ 1.1	333	83.25
> 1.1	67	16.75
Urea(BUN) in mg/dl		
≤ 50	339	84.75
> 50	61	15.25
Uric acid(in mg/dl)		
≤ 7.1	342	85.5%
> 7.1	58	14.5%

**Table 5:** Multivariable ordinal logistic regression for determinants of HR attending ophthalmic centers in Northern Ethiopia, 2025

Variables		COR 995% CI)	AOR (95% CI)	*p*-value
History of cataract surgery	Yes(103)	3.00(1.91–4.69)	2.19 (1.32, 3.61)	**0.002**
	No (297)	1	1	
Physician Advice	Yes (179)	0.72(0.47–1.11)	0.81(0.47, 1.43)	0.412
	No (221)		1	
Diabetes mellitus	Yes (211)	2.09(1.43–3.06)	1.61 (1.05, 2.47)	**0.031**
	No (189)	1	1	
CKD	Yes (77)	1.63(1.04–2.54)	3.16 (1.82, 5.48)	**0.0001**
	No (323)	1	1	
Sex	Male (223)	1.15(0.78–1.68)	1.31 (0.77, 1.67)	0.55
	Female (177)	1	1	
Regular HTN checkup	Yes (332)	0.56(0.33–0.96)	0.47 (0.27, 0.80)	**0.006**
	No (68)	1	1	
Alcohol drinking	Yes (47)	0.63(0.36–1.10)	0.73 (0.41, 1.29)	0.261
	No (357)	1	1	
Eye examination after HTN diagnosis	Yes (164)	1.51(1.03–2.23)	0.44 (0.25, 0.77)	0.78
	No (236)	1	1	
/cut1		-2.38	-3.03-1.73	
/cut2		-0.3391492	-0.94-0.26	
/cut3		2.94	2.23–3.65	

## Data Availability

All participant data generated and/or analyzed during this study are available from the principal investigator upon reasonable request.

## Abbreviations

AOR: Adjusted Odd Ratio
BP: Blood Pressure
CI: Confidence Interval
CKD: Chronic Kidney Disease
D: Diopter
HR: Hypertensive Retinopathy
HTN: Hypertension
IQR: Inter Quartile Range
KG: Kilogram
MmHg: Millimeter of Mercury
TOD: Target Organ Damage
VIF: Variance Inflation Factor
